# ZumBeat: Evaluation of a Zumba Dance Intervention in Postmenopausal Overweight Women

**DOI:** 10.3390/sports4010005

**Published:** 2016-01-25

**Authors:** Anja Rossmeissl, Soraya Lenk, Henner Hanssen, Lars Donath, Arno Schmidt-Trucksäss, Juliane Schäfer

**Affiliations:** 1 Division of Sports and Exercise Medicine, Department of Sport, Exercise and Health, University of Basel, Birsstrasse 320 B, Basel 4052, Switzerland; soraya.lenk@unibas.ch (S.L.); henner.hanssen@unibas.ch (H.H.); juliane.schaefer@unibas.ch (J.S.); 2 Division of Movement and Exercise Science, Department of Sport, Exercise and Health, University of Basel, Birsstrasse 320 B, Basel 4052, Switzerland; lars.donath@unibas.ch; 3 Basel Institute for Clinical Epidemiology and Biostatistics, University Hospital Basel, Spitalstrasse 12, Basel 4031, Switzerland

**Keywords:** menopause, dance, cardiorespiratory fitness, Zumba, barriers, quality of life

## Abstract

Physical inactivity is a major public health concern since it increases individuals’ risk of morbidity and mortality. A subgroup at particular risk is postmenopausal overweight women. The aim of this study was to assess the feasibility and effect of a 12-week ZumBeat dance intervention on cardiorespiratory fitness and psychosocial health. Postmenopausal women with a body mass index (BMI) >30 kg/m^2^ or a waist circumference >94 cm who were not regularly physically active were asked to complete a 12-week ZumBeat dance intervention with instructed and home-based self-training sessions. Before and after the intervention, peak oxygen consumption (VO_2peak_) was assessed on a treadmill; and body composition and several psychometric parameters (including quality of life, sports-related barriers and menopausal symptoms) were investigated. Of 17 women (median age: 54 years; median BMI: 30 kg/m^2^) enrolled in the study, 14 completed the study. There was no apparent change in VO_2peak_ after the 12-week intervention period (average change score: −0.5 mL/kg/min; 95% confidence interval: −1.0, 0.1); but, quality of life had increased, and sports-related barriers and menopausal symptoms had decreased. A 12-week ZumBeat dance intervention may not suffice to increase cardiorespiratory fitness in postmenopausal overweight women, but it increases women’s quality of life.

## 1. Introduction

Many physiologic changes are brought about during menopause and the shifting levels of hormones, associated with a considerable increase in cardiovascular risk [1,2]. In addition to the health hazard associated with the metabolic changes and increasing abdominal fat commonly concurring with menopause, many women experience weight gain as a function of aging [3,4]. The weight gain is partly explained by decreasing basal metabolic rates and decreasing energy expenditure due to diminished physical activity [5,6]. Therefore, the observed decline in physical activity around the age of 55 years is a crucial issue in the postmenopausal phase [7,8], since inactive postmenopausal women who are overweight or obese have a substantially increased risk for cardiovascular diseases [9].

Much research has documented the tremendous benefits of physical activity on various health aspects [10]. Previous studies have additionally enhanced our understanding for the causes of inactivity and identified various categories of barriers associated with inactivity behavior in women at midlife [11,12,13]. Dance, on the other hand, is the second most popular leisure time physical activity after walking in women across all ages (25 to 75 years plus) [14]. Convincing benefits of dancing activities on physiologic, endocrine, cognitive and psychological levels have previously been shown [15,16,17,18,19,20,21]. Several studies revealed improved fitness, weight loss, reductions in cholesterol levels or inflammatory markers in women at risk after various forms of aerobic dancing [18,22,23,24,25,26,27], including “aerobics”, “step aerobics” and “cultural dances”. Zumba as a type of fitness dance that combines Latin rhythms and aerobics has rapidly reached tremendous popularity lately [28]. The first studies on Zumba revealed its sufficiency as a training method that is able to enhance cardiorespiratory fitness [29,30]. Moreover Zumba Gold has been shown to be safely applied in elderly or even chronically-ill people [31,32], although two studies warn of possible injuries associated with Zumba fitness and the wrong footwear [33,34]. In young normal weight females, Zumba improves fitness, trunk strength endurance, balance and quality of life [35]. Furthermore, Zumba has been shown to reduce neck-shoulder pain, as well as fat mass and to improve aerobic fitness in the setting of a workplace intervention [36,37,38]. A pilot study conducted at the same time as our study in a population of middle-aged obese women with metabolic syndrome reported weight reductions of 2.07 pounds on average, as well as improvements in systolic and diastolic blood pressure and fasting triglycerides after a 12-week intervention [39]. Quite recently, studies also started to examine aspects of motivation, self-perceived fitness and autonomy [40,41]. One of these studies published in 2015 found enhanced intrinsic motivation associated with fitness improvements, as well as reductions in body weight and fat mass after 16 weeks of Zumba dance in obese middle-aged women [40]. However, there is still a paucity of data on physical and mental health benefits from Zumba-style dancing activities in the older, sedentary overweight population. The aim of this study was to assess the feasibility of a 12-week ZumBeat dance intervention in sedentary, postmenopausal overweight women and to generate preliminary effect size estimates regarding cardiorespiratory fitness (primary outcome) and anthropometric and psychometric parameters (secondary outcomes).

## 2. Materials and Methods

### 2.1. Study Design

In this single-arm, monocentric, prospective interventional study, participants were examined before and after a 12-week Zumba dance intervention (ClinicalTrials.gov Identifier: NCT02384694). The 12-week intervention comprised a total of three planned weekly training sessions. Participants were asked to attend at least 2 out of 3 weekly sessions offered in-person at the facilities of the University of Basel. In addition, they were asked to complete at least one weekly session as home-based training. The study was approved by the local ethics committee (Ethikkommission Nordwest-und Zentralschweiz, Basel, Switzerland). Written informed consent was obtained from all participants prior to the start of the study.

### 2.2. Participants

Sedentary women were recruited from the community through leaflet advertisements in doctor’s offices and pharmacies (700 pieces), on online marketplaces, University pin boards and during public talks. Moreover, heads of local clinics (e.g., endocrinology, internal medicine) were contacted via e-mail as mediators to address possible candidates. Potential participants contacted the study staff and were screened for eligibility. The inclusion criteria were: (1) postmenopausal woman between 45 and 65 years of age; (2) body mass index (BMI) >30 kg/m^2^ or waist circumference >94 cm; and (3) not regularly physically active more than once a week. Subjects were excluded, when they: (1) were presently enrolled in another research study; (2) were unable to attend the training sessions; or (3) had medical contraindications for exercise (such as pulmonary and/or cardiovascular disease, epilepsy or an elevated risk of falls or other limitations for safe study participation). Women with renal dysfunctions, active malignancies or recent chemotherapies (<6 months) were also excluded. Contraindications for exercise were determined via assessment of medical history and clinical examination. The entire enrolment, intervention and measurement phase took 6 months. The study was conducted between May and October 2014. Participants were asked to maintain their usual dietary habits.

### 2.3. Measurements

At the initial visit, participants were interviewed and examined by an experienced physician. Anthropometric and psychometric parameters were collected before the exercise testing.

Demographic and anthropometric data, including height (in cm, stationary device, without shoes), weight (in kg, Inbody 720^®^ Bioimpedanzmessgerät (JP Global Markets GmbH, Eschborn, Germany), in underwear), waist circumference (in cm, with tape directly above the iliac crest) and calculated BMI (in kg/m^2^), were obtained. Blood pressure and heart rate at rest were recorded using an automated oscillometric device (Mobil-O-Graph NG, I.E.M., Stolberg, Germany) after 10 min of rest in supine position. The cuff was inflated 3 times to supra-systolic values (30 mmHg above the systolic blood pressure) automatically with a succeeding pressure release at a rate of 3 mmHg/ second.

We compiled a test battery consisting of common tests for the assessment of facets of psychosocial and physical health in association with being overweight, eating behavior, depression, menopausal symptoms, subjective health and quality of life, as well as sports-related barriers (Supplementary Materials, Table S1).

### 2.4. Exercise Testing

**Figure 1:**
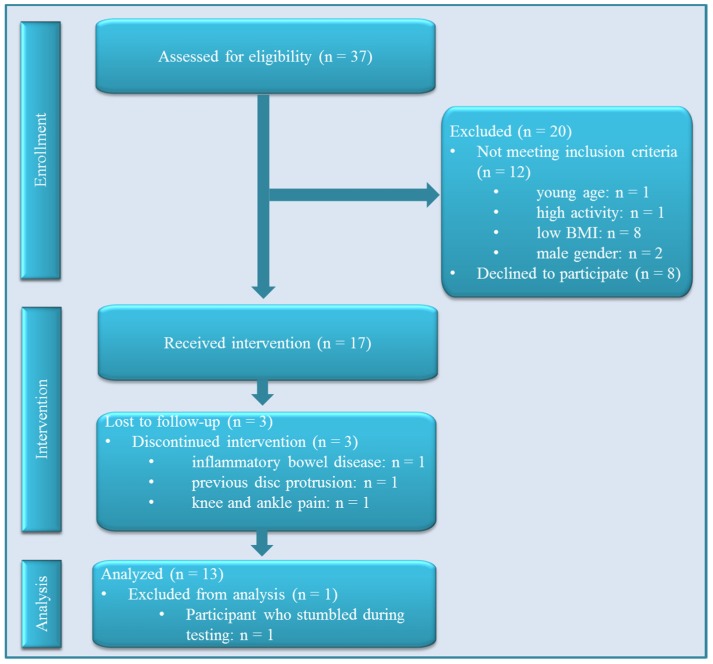
Flow of participants through the trial. Abbreviations: BMI, body mass index.

Peak oxygen consumption (VO_2peak_) and maximum heart rate (HR_max_) were assessed during spiroergometric cardiopulmonary exercise testing on a treadmill (H/P/Cosmos Pulsar 2005, H/P Cosmos Sports and Medical GmbH, Nussdorf-Traunstein, Germany) equipped with a suspension system and an emergency stop as routine safety measures. Participants were allowed to use handrails in case of instability and advised to look straight ahead during the testing. The space behind the treadmill was three meters; no sharp surfaces or material were in reach of the treadmill. One participant stumbled while stepping partly off the running belt, and the treadmill was stopped immediately (see flow chart Figure 1). As a consequence, safety measures were formally reinforced prior to the testing of the following participants. Before each exercise test, the spirometry system (Cortex Metalyzer^®^ 3B (Cortex Biophysik GmbH, Leipzig, Germany)) was calibrated by two-point gas calibration with gases of known composition. After a 5-min period of familiarization and safety instruction while standing on the treadmill, participants performed a graded exercise test (pepper ramp protocol [42]). Throughout the entire duration of the test, cardiac function was recorded on a 12-lead electrocardiogram (custo cardio 100, CustoMed, Ottobrunn, Germany) under the supervision of a staff physician. Peak blood pressure values were obtained to rule out hypertensive blood pressure. To determine subjective effort, ratings of perceived exertion were acquired (Borg Scale [43]). The test was terminated upon reaching volitional exhaustion or fulfilment of absolute cardiorespiratory exhaustion criteria (2 or more out of the following: heart rate >80% of the predicted HR_max_ (= (220-age) ± 10%), ratings of perceived exertion ≥18 (Borg scale: 6 to 20), respiratory exchange ratio ≥1.05, no further increment in VO_2peak_, supervisor’s impression).

### 2.5. Dance Intervention

We modified the original higher impact Zumba-style and composed a set of ZumBeat choreographies aiming at a reduced strain for the musculoskeletal system by avoiding high impact jumping movements, appropriate for an overweight population (Supplementary Materials, Figures S1 and S2), similar to Zumba Gold^®^. During the 12-week intervention period, 3 instructed 60-min classes were provided per week, of which participants were instructed to attend a minimum of 2. Participants were allowed to join the in-person training up to three times per week and, thus, compensate for missed trainings in earlier or later weeks. Positive feedback and verbal encouragement were used to enhance adherence during the in-person sessions. Participants also received a DVD with dance moves, recorded by our team, for the home-based self-trainings. Participants were instructed to perform home-based self-trainings at least once a week by choosing from a set of 10 dances on a menu plus additional warm-up and cool-down sections. Self-monitoring is considered the cornerstone of behavioral obesity treatment as it enhances self-awareness and self-regulatory capabilities [44,45]. Although a causal relationship has not been established, the literature suggests that consistent self-monitoring of exercise is associated with a greater amount of exercise and weight loss, as well as fewer difficulties with exercise [46]. We therefore instructed our participants to record their home-based training sessions on a log.

During 3 instructed training sessions in Weeks 1, 5 and 12, heart rate was recorded on a Polar watch (RS 400, Polar Electro Oy, Kempele, Finland) during the whole course of the session. Training heart rates were calculated from dancing periods only (40 to 45 min).

### 2.6. Statistical Analyses

The primary outcome of this study was the change in VO_2peak_ (in mL/kg/min) after the 12-week intervention period; secondary outcomes were changes in BMI, weight, waist circumference, percent body fat, visceral fat mass, muscle mass, systolic blood pressure, diastolic blood pressure, resting heart rate, quality of life, sports-related barriers, menopausal symptoms, depression, psychiatric symptoms, impulsivity and eating behavior. Paired *t*-tests were used to assess changes in outcome after the intervention period. In sensitivity analyses, we removed a few unusual data points to see whether our analyses were robust to outliers. For each analysis, we report the estimated change in outcome with its 95% confidence interval in order to emphasize clinical relevance over statistical significance. To facilitate use of our data for planning future studies, we also report Cohen’s d as an effect size measure, which can be readily interpreted as the percentage of the standard deviation of the change scores (such that a Cohen’s d of 0.5 means the difference equals half a standard deviation). We used R Version 3.1.2 (R Foundation for Statistical Computing, Vienna, Austria) and the R add-on package lattice Version 0.20–30 for our analyses and graphics [47].

### 2.7. Power Calculation

Based on previous literature [18,22,25,48,49], we assumed a mean VO_2peak_ of 25 to 35 mL/kg/min in women between 45 and 65 years of age, a standard deviation of 5 mL/kg/min and a correlation between baseline and follow-up VO_2peak_ of 0.7. A 3.5-mL/kg/min (= 1 MET, metabolic equivalent) higher fitness level was associated with a 13% risk reduction of all-cause mortality and a 15% lower risk for cardiovascular disease events or death in men and women according to epidemiological data [50]. A large scale longitudinal study observed a risk reduction of 15% all-cause and 19% cardiovascular mortality with each MET increment in fitness [51]. Based on these previous findings, we aimed at a risk reduction of at least 10% for cardiovascular disease events and, therefore, considered an improvement in VO_2peak_ of 3 mL/kg/min as clinically meaningful. With 17 study participants, the power was 85% to detect such improvement (using a paired *t*-test at a 2-sided significance level of 0.05).

## 3. Results

### 3.1. Participant Characteristics

Thirty-seven subjects responded to our advertisements. Twenty of them did not start the intervention because of failure to meet the eligibility criteria or refusal to participate (Figure 1). Seventeen overweight women were enrolled in the study, with a mean age of 55 years, median BMI of 30 kg/m^2^ and median waist circumference of 103 cm (Table 1). Out of the 17 participants, 13 were on oral medications; six took antihypertensive drugs (two β-blockers); two antidepressants; one a long-acting β_2_ agonist.

**Table 1:** Participant baseline characteristics.

Characteristic	All Participants (*n* = 17)
Female sex, *n*	17
Age, years	55 (6)
Height, cm	167 (9)
Weight, kg	85 (79, 94)
BMI, kg/m^2^	30 (29, 33)
Waist circumference, cm	103 (102, 114)
Percent body fat, %	39 (8)
Visceral fat mass ^1^, cm^2^	130 (118, 156)
Systolic blood pressure ^2^, mmHg	126 (12)
Diastolic blood pressure ^2^, mmHg	80 (10)
Smoking, n	–
Non-smoker	13
Ex-smoker	3
Current smoker	1

Abbreviations: BMI, body mass index; data are the mean (standard deviation) or median (interquartile range) if not stated otherwise; ^1^ Available in 16/17 participants; ^2^ after excluding one participant who forgot to take relevant medication on the day of the baseline visit, available in 16/17 participants.

### 3.2. Training Completion and Intensity

Out of the 17 participants, three were lost to follow-up. The remaining 14 participants attended a median of 20 of the recommended 24 instructed training sessions (interquartile range (IQR) 14, 24) and completed a median of seven of the recommended 12 home-based self-training sessions (IQR 4, 9), where one participant did not provide information on the number of completed home-based trainings. The overall median training performance was 30 of the recommended 36 training sessions (IQR 20, 34). In detail: two participants completed eight and 12 instructed sessions (corresponding to an average of ≤1 attendance per week); nine completed between 13 and 24 instructed sessions (corresponding to an average of one to two attendances per week); and three participants completed 30 instructed sessions (corresponding to an average of >2 attendances per week). Regarding home-based self-training, there were 10 participants that completed between two and nine sessions (corresponding to an average of <1 completion per week); two completed 18 sessions (corresponding to an average of 1.5 completions per week); and one participant completed 20 sessions (corresponding to an average of >1.5 completions per week). Overall, five participants completed between eight and 24 instructed or home-based self-training sessions (corresponding to an average of ≤2 trainings per week); six completed between 25 and 34 sessions (corresponding to an average of two to three trainings per week); and three participants completed between 37 and 41 sessions (corresponding to an average of >3 sessions per week).

**Figure 2:**
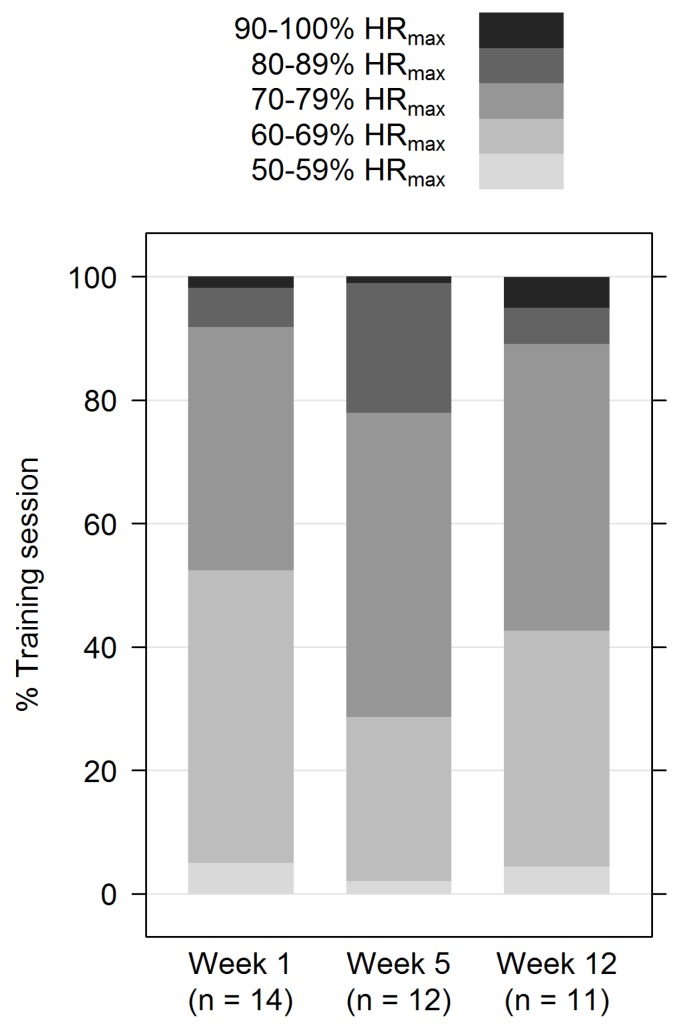
Average percentage of time spent at certain heart rate zones during three (instructed) training sessions in Weeks 1, 5 and 12. Heart rate zones were calculated individually based on the maximum heart rate (HR_max_) during baseline exercise testing.

During the instructed training sessions in Weeks 1, 5 and 12, participants spent ≤5% of the time at 50% to 59% of a participant’s individual HR_max_, while the remaining time of the training sessions was spent at heart rate zones ≥60% of the HR_max_ (Figure 2). Participants performed the instructed training sessions in Weeks 1, 5 and 12 at a median of 69% (IQR 65, 74), 75% (IQR 69, 78) and 72% (IQR 67, 74) of the HR_max_ (Supplementary Materials, Figure S3).

### 3.3. Effect of the Intervention on Cardiorespiratory Fitness

**Figure 3:**
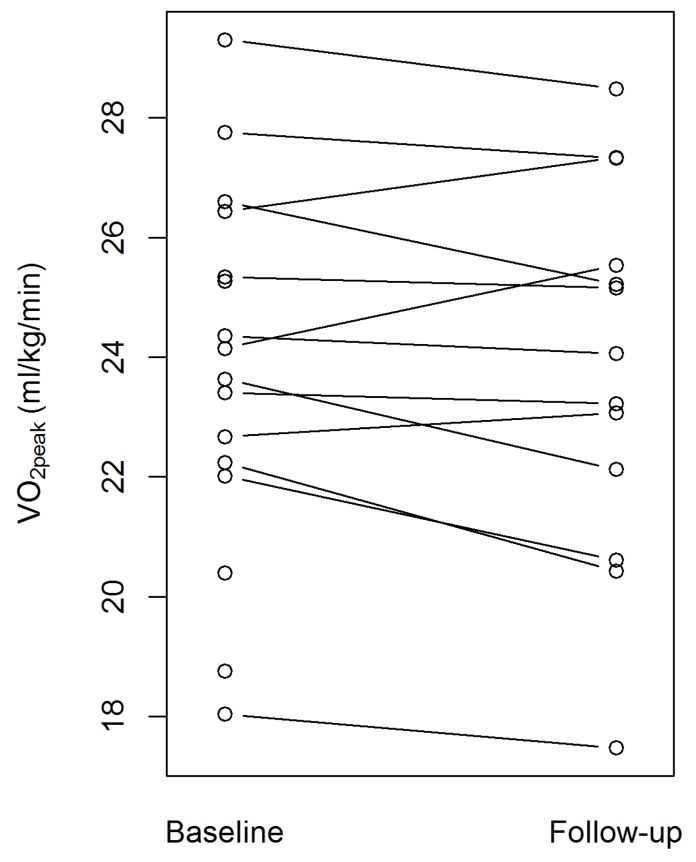
Individual responses in peak oxygen consumption (VO_2peak_) showing the direction of change.

One participant stumbled during treadmill testing and did not complete the test protocol. We therefore excluded this participant from the analysis of both the VO_2peak_ and HR_max_. For each participant considered for analysis, Figure 3 shows the change in VO_2peak_ after the 12-week intervention period. On average, there was no apparent change in VO_2peak_ (−0.5 mL/kg/min, 95% confidence interval (CI) −1.0, 0.1; *p* = 0.114, Table 2), where all participants considered for analysis fulfilled at least two objective exertion criteria at baseline and follow-up testing.

**Table 2:** Effect of the 12-week ZumBeat dance intervention on cardiorespiratory fitness, anthropometry and cardiovascular parameters.

Parameter	N ^1^	Intervention (*n* = 17)		Change from Baseline (95% CI)	*p*-Value	Cohen’s d
		Baseline (Mean (SD))	Follow-up (Mean (SD))			
VO_2peak_, mL/kg/min	13	24.3 (2.9)	23.9 (3.2)	−0.5 (−1.0, 0.1)	0.114	–0.47
HR_max_, bpm	12	161.8 (15.5)	164.5 (17.0)	2.7 (−2.8, 8.1)	0.305	0.31
***Anthropometry***						
Weight, kg	14	91.0 (13.1)	90.4 (11.6)	−0.6 (−2.4, 1.3)	0.509	–0.18
BMI, kg/m^2^	14	32.7 (4.9)	32.5 (4.2)	−0.2 (−0.9, 0.4)	0.444	–0.21
Waist circumference, cm	14	108.8 (9.2)	107.2 (8.5)	−1.6 (−3.3, 0.2)	0.072	–0.52
Percent body fat, %	14	39.5 (8.2)	38.1 (7.4)	−1.3 (−3.6, 0.9)	0.210	–0.35
Fat mass, kg	14	36.1 (10.2)	34.7 (9.0)	−1.4 (−3.7, 0.9)	0.200	–0.36
Visceral fat mass, cm^2^	13	140.7 (25.6)	138.4 (27.1)	−2.2 (−9.6, 5.1)	0.523	–0.18
Muscle mass, kg	14	30.3 (5.3)	31.0 (5.0)	0.7 (−0.3, 1.7)	0.162	0.40
***Cardiovascular parameters***						
SBP, mmHg	12	126.6 (14.2)	131.0 (12.3)	4.4 (−1.4, 10.2)	0.124	0.48
DBP, mmHg	12	80.4 (9.9)	83.3 (9.8)	2.9 (−1.2, 6.9)	0.146	0.45
Resting heart rate, bpm	13	63.5 (7.5)	62.3 (6.5)	−1.2 (−5.5, 3.2)	0.578	–0.16

Abbreviations: VO_2peak_, peak oxygen consumption; HR_max_, maximum heart rate; BMI, body mass index; SBP, systolic blood pressure; DBP, diastolic blood pressure; SD, standard deviation; CI, confidence interval; ^1^ Number of participants with baseline and follow-up data available.

### 3.4. Effect of the Intervention on Anthropometric Parameters

On average, anthropometric parameters showed small changes in the expected direction, though associated with considerable uncertainty (Table 2). Data on visceral fat mass were only available in 13 participants at baseline and follow-up. There were no changes in medication between the baseline and follow-up visit, except for two women who forgot to take their antihypertensive medications at the day of either the baseline or follow-up visit and who were therefore excluded from the blood pressure analyses. One of them was also excluded from the heart rate analyses (β-blocking agents) (Table 2).

### 3.5. Effect of the Intervention on Psychometric Parameters

#### 3.5.1. Quality of Life

At the end of the 12-week intervention period, participants’ overall self-rated quality of life had increased by 9.2 out of 100 points (95% CI 1.6, 16.8) on the Impact of Weight on Quality of Life (IWQOL) questionnaire [52]; and self-esteem and work-related quality of life had increased by 15.6 points (95% CI 2.8, 28.4) and 10.3 points (95% CI 0.1, 20.4) (Table 3).

**Table 3:** Effect of the 12-week ZumBeat dance intervention on psychometry.

Questionnaire	N ^1^	Intervention (*n* = 17)		Change from Baseline (95% CI)	*p*-Value	Cohen’s d	+/− ^2^	Norm ^3^
		Baseline (Mean (SD))	Follow-up (Mean (SD))					
IWQOL (0–100)	14	−	−	−	−	−	−	−
Total score	−	79.4 (17.2)	88.7 (8.8)	9.2 (1.6, 16.8)	0.022	0.70	+	91.8
Physical function	−	75.5 (17.9)	83.6 (13.3)	8.1 (−1.2, 17.4)	0.082	0.50	+	90.0
Self-esteem	−	68.1 (29.0)	83.7 (19.5)	15.6 (2.8, 28.4)	0.021	0.70	+	87.5
Sexual life	−	86.2 (25.4)	92.9 (18.2)	6.7 (−2.0, 15.3)	0.119	0.45	+	95.1
Public distress	−	91.9 (14.7)	96.1 (5.3)	4.2 (−2.3, 10.7)	0.189	0.37	+	96.5
Work	−	87.5 (18.0)	97.8 (5.2)	10.3 (0.1, 20.4)	0.048	0.58	+	95.4
SBB (0 to 4)	14	2.5 (0.5)	2.1 (0.6)	−0.4 (−0.7, −0.2)	0.001	−1.09	+	2.2
MRS(0 to 44)	14	11.9 (7.6)	9.5 (7.1)	−2.4 (−4.6, −0.2)	0.036	−0.62	+	8.8
BDI (0 to 63)	14	7.2 (7.8)	4.8 (5.3)	−2.4 (−5.6, 0.7)	0.118	−0.45	+	7.7
ISR (0 to 4)	14	0.5 (0.5)	0.4 (0.4)	−0.1 (−0.2, 0.0)	0.216	−0.35	+	0.4
I-8 (0 to 4)	14	−	−	−	−	−	−	−
Urgency	−	2.3 (0.6)	2.2 (0.8)	−0.1 (−0.4, 0.1)	0.314	−0.28	+	2.5
Intention	−	3.6 (1.0)	4.0 (0.6)	0.4 (−0.1, 0.9)	0.094	0.48	+	3.8
Endurance	−	4.1 (0.6)	3.9 (0.9)	−0.2 (−0.6, 0.2)	0.254	−0.32	−	4.3
Risk	−	3.6 (0.8)	3.2 (0.8)	−0.4 (−0.9, 0.2)	0.156	−0.40	+	2.8
FEV	14	−	−	−	−	−	−	−
Dietary restraint (0 to 21)	−	9.1 (3.8)	9.8 (4.4)	0.7 (−2.0, 3.4)	0.582	0.15	+	8.2
Disinhibition (0 to 16)	−	8.2 (3.6)	7.1 (3.2)	−1.1 (−2.5, 0.4)	0.132	−0.43	+	7.1
Hunger (0 to 14)	−	7.2 (3.8)	5.0 (3.7)	−2.2 (−4.2, −0.3)	0.028	−0.66	+	5.7

Abbreviations: IWQOL, Impact of Weight on Quality of Life; SBB, Sports-related Situational Barriers Scale; MRS, Menopause Rating Scale; BDI, Beck Depression Inventory; ISR, ICD-10-Symptom-Rating; I-8, German Version of the UPPS Impulsive Behavior Scale; FEV, German Version of the Dutch Eating Behavior Questionnaire (DEBQ); SD, standard deviation; CI, confidence interval; ^1^ Number of participants with baseline and follow up data available; ^2^ ”+” symbolizes improvement of the test result, “−“ symbolizes deterioration; ^3^ normative values for the general population.

#### 3.5.2. Menopausal Symptoms

Symptoms that are frequently encountered during menopausal transition had decreased by 2.4 out of 44 points (95% CI 0.2, 4.6) on the Menopause Rating Scale (MRS) [53] (Table 3).

#### 3.5.3. Sports-Related Barriers

Barriers towards exercise had decreased by 0.4 out of four points (95% CI 0.2, 0.7) on the Sports-related Situational Barriers (SBB) scale [54] (Table 3).

#### 3.5.4. Psychiatric Symptoms

Levels of depression on the Beck Depression Inventory II (BDI) had improved slightly [55]. Other psychiatric complaints, such as anxiety, compulsion, somatization or eating disorders, were not prevalent among the study participants and were essentially unchanged after the end of the intervention period, based on the results of the ICD-10 Symptom Rating (ISR) scale [56] (Table 3).

#### 3.5.5. Impulsivity and Eating Behavior

While most impulsivity sub-scales were essentially unchanged after the end of the intervention period, intentional behavior had increased slightly, with an average change from baseline of 0.4 out of four points (95% CI −0.1, 0.9) on the I-8 questionnaire (German Version of the UPPS Impulsive Behavior Scale) [57]. Dietary restraint and disinhibition towards food had not changed after the end of the intervention period. However, feelings of hunger were reduced by 2.2 out of 14 points (95% CI 0.3, 4.2) on the German Version of the Dutch Eating Behavior Questionnaire [58] (Table 3).

### 3.6. Sensitivity Analyses

Compared to the main analysis of the VO_2peak_, exclusion of two participants who stopped exercise testing prior to having reached (subjective) exertion led to a similar pattern of change after the 12-week intervention period (average change score: −0.2 mL/kg/min, 95% CI −0.8, 0.3; *p* = 0.382). Estimated changes from baseline were generally decreased after excluding a few outliers with unusually strong improvements in outcome (Supplementary Materials, Table S2). For example, changes in percent body fat and muscle mass were decreased after excluding two outliers with an unusually strong decrease in percent body fat and one outlier with an unusually strong increase in muscle mass, respectively.

## 4. Discussion

Developing, piloting, evaluating, reporting and implementing are important steps for interventions, as suggested by the Medical Research Council [59]. Generally, studies can be declared as feasible when they fulfil the criteria of successful recruitment, retention/compliance/adherence and safety.

With regard to feasibility assessment in the process of evaluating and developing a future intervention, one result of this study is that the intervention itself appears to be feasible: in our study, only three participants (equaling 18%) discontinued the study participation. Previous literature suggests drop-out rates due to orthopedic problems of 25% [41]. We attribute the relative success to the low hazardous style of the ZumBeat dance moves. Most Zumba classes demand high coordination and experience with the choreographies. Assuming a lack of the above, the likelihood for musculoskeletal injury is increased. The small number of complications from the training, the small drop-out rate and good adherence in this study suggest the feasibility of the intervention itself.

We observed no benefit for cardiorespiratory fitness following a 12-week ZumBeat dance intervention in postmenopausal overweight women and only small benefits, if any, for body composition. However, single items of health-related quality of life may be enhanced and menopausal symptoms and sports-related barriers reduced. This implies possible reductions of problems often encountered in the field of activity promotion and might contribute to a better understanding in this important public health area. Future projects should implement and thoroughly assess this topic.

The literature suggests that Zumba dance is a suitable training method to enhance cardiovascular fitness and strength [29,35,38]. However, older, overweight populations have rarely been studied. A recent publication by Dalleck *et al.* shows that Zumba Gold^®^ training can be categorized as moderate exercise and is in line with current guidelines for improving and maintaining cardiorespiratory fitness [60]. While on average, women in this study did not improve their VO_2peak_, Krishnan *et al.* found an improvement in VO_2peak_ of 1 mL/kg/min in a similar study population after a 16-week, three times weekly, 60-min per session Zumba intervention [40]. In their study, VO_2peak_ was not measured, but calculated after conducting a walking test. A quality criterion for our study is the direct measurement of VO_2peak_. Another study among female hospital workers indicated an improvement in VO_2peak_ of 1.46 mL/kg/min (95% CI 0.17, 2.75) after 12 weeks of training and a reduction in fat mass comparable to that observed in our study [60]. However, the participants were younger (mean age: 46 years), had a lower BMI (mean BMI: 26 kg/m^2^) and, therefore, might have adapted to the training more easily. Moreover, this might have allowed them to exercise at greater training intensities right from the start. The reported change in VO_2peak_ corresponds to improvements between 0.9% and 9%, 5% on average only. This still lies in the range possible of retest adaption effects [61] or corresponds to a 3% to 5% reduction of the relative risk for cardiovascular events [51]. The great variability in study participation with a lack of continuity of exercise over the summer period in our study may explain why we were not able to add to the growing body of evidence for an effect of Zumba on fitness. This might have diminished measurable improvements and be explanatory for the missing increment in VO_2peak_, but clearly reflects real-life circumstances. The endurance section of the lessons (excluding warm-up and cool-down) evoked a rise in heart rates above the level necessary to acquire training adaptions and gains in fitness, with median heart rates ranging between 69% and 75% of the HR_max_ (Supplementary Materials, Figure S3) and the main portion of the lesson at heart rate zones between 60% and 79% of the participants’ HR_max_ (Figure 2). Exercise intensity progressed from moderate to higher intensities over the time-course of the intervention, as recommended in exercise training guidelines [62]. According to the subjective impression of the instructor, the training sessions were all well tolerated. This confirms that the intensity of the training itself meets current recommendations and, therefore, should have been sufficient to positively influence fitness [63]. Moreover, a pilot study suggests that Zumba^®^ dance classes might allow greater energy expenditure, but Zumba^®^ DVD workouts might be suitable to maintain fitness [64].

On average, participants did not show a large benefit in anthropometric parameters after the 12-week intervention period. Single participants, however, showed extreme changes in body composition, with one participant losing 7.4% and another losing 10.8% body fat. There was no evidence for a change in blood pressure, but both systolic and diastolic average levels in this small sample appeared slightly higher after the intervention. This is in line with results from an eight-week Zumba^®^ intervention, which did not result in relevant changes in body composition [41]. A recent publication by Cugusi *et al.* showed significant weight and fat mass reductions, as well as gains in muscle mass after a 12-week Zumba dance intervention similar to ours, in a younger overweight population (mean age 38.9 ± 9.7 years) [65]. They also reported decreases in blood pressure levels. This indicates that Zumba may contribute to significant improvement in body composition, but whether this is possible in an elderly overweight population with appropriate training time and effort remains to be proven.

Cugusi *et al.* investigated quality of life in relation to Zumba dancing [65]. In their study, physical functioning and emotional role were the only two out of eight domains assessed that showed significant amelioration. Our study suggests improvements in quality of life scores associated with being overweight. At baseline, scores were ranging between normal values for healthy people and participants in weight reduction programs in each subscale. After the intervention, the scores appeared improved, but a causal attribution to the intervention cannot be made due to the study design. Larger scaled controlled trials are needed for confirmation.

Our results suggest a reduction in sports-related barriers (0.4 out of four points (95% CI 0.2, 0.7) on the SBB scale). Sedentariness implies the existence of manifold barriers and a lack of motivation. One major factor accounting for inactivity is the lack of self-motivation. Greater self-efficacy, on the other hand, has been identified as key determinant in increasing physical activity [13,66]. Studies show that a recent exercise experience or mastery can improve self-efficacy beliefs and increase exercise adherence in the maintenance phase [67]. Therefore, an increase in self-esteem and a decrease in barriers as observed in this study could be a first step towards greater activity participation. Future controlled studies are required to confirm our exploratory results regarding barriers and should simultaneously monitor associated self-motivation and self-efficacy beliefs.

Average baseline values of all sub-scores and the total MRS score were above the healthy European norm [68]. While previous research has revealed ambiguous results of exercise on menopausal symptoms, menopausal symptoms seemed lower post-intervention in this study and may be worth future exploration.

Two participants showed elevated levels of depression on the BDI-II at baseline, beyond the cut-off for moderate depression; both of them had reduced their depression scores after the end of the intervention (one of them by more than eight points, representing a clinically-relevant improvement). Average depression levels in our study were not elevated and remained essentially unchanged after the intervention, whereas other studies have reported higher depression levels among diabetic and obese people [69,70,71,72]. Furthermore, we did not find symptoms suggestive of other psychiatric disorders nor did they change over the course of the intervention, but due to the small sample size and single-arm design, a generalization of these results in not feasible.

While impulsivity as a construct is strongly associated with behavioral control and adaptive regulations to the individual’s surroundings, it is often more pronounced in patients with binge eating disorders or obesity [73]. Physical activity, on the other hand, has been shown to moderate impulsive behavior in addition to behavioral therapy [74]. In this study, impulsive behavior was more prevalent at baseline compared to normative values on all four sub-scores of the I-8 questionnaire, but did not seem to be affected by the intervention.

In addition, this study revealed encouraging results suggesting a reduction in hunger perceptions, which could lead to a more stable eating behavior, but still needs to be proven through further research.

### Limitations

This study presents some limitations. First, due to recruitment problems, our study has a small sample size and lacks a control group. We originally designed this study as a randomized controlled trial, but were only able to recruit 17 participants and, therefore, changed the study design, with approval from the ethics committee, to a prospective, interventional single-arm study. Despite our great efforts in terms of recruitment, we were not able to meet the target total sample size as determined for a two-arm trial, including a control group. Interested women were too young or active, while the targeted participants did not respond to our advertisements. The advertisements might have been more successful if more research personnel had approached potential candidates verbally in person or via local media. We were unable to place costly advertisements in newspapers or distribute leaflets to private households for this study, as it was only funded by internal resources. Moreover, future studies might consider a matching of the contact person with participants (older, overweight woman), a more convenient location and closer telephone follow-up in order to enhance recruitment efficiency [66]. In addition, incentives for study participation should be reconsidered. Given the low response to advertisement in this study and even lower eligibility rates, careful reconsideration of the strict inclusion criteria might be another approach, but simultaneously changes the purpose of the study. Our practical experience is comparable to the Zumba pilot study reported by Araneta *et al.* in which 35 women completed eligibility screening by phone, 23 were eligible for a first visit, 18 met eligibility criteria, 16 initiated Zumba classes and 13 completed the study. Therefore, these response and retention rates may be characteristic for this type of study in a similar population. Our recruitment strategies were insufficient to generate a sample large enough for a randomized controlled trial, and therefore, this part of the project was not feasible. Recruitment strategies need to be carefully reconsidered before starting a large randomized controlled trial. This may include the option of multi-center studies. With the above-mentioned limitation (lacking a control group) in mind, all resulting data and their interpretations below need to be handled with caution. They should rather be interpreted as exploratory indicators serving to generate effect sizes for future controlled trials.

Secondly, the study has a short length of follow-up, limiting the strength of findings on the mid- to long-term effects of the ZumBeat dance intervention.

Thirdly, participants who dropped out of the intervention could not be included in the final results, raising issues of effectiveness among those who stop exercising.

In sum, our study suggests a decrease in sports-related barriers, better attitudes towards exercise and improvements in weight-adjusted quality of life following a 12-week ZumBeat dance intervention. As a result, individuals may be more active in the post-intervention period, so that cardiorespiratory fitness and weight are then steadily improved. This has important public health implications, since current literature suggests that postmenopausal sedentary women who are overweight have an increased cardiovascular risk, which can be modified by physical activity [9]. A short-term ZumBeat dance intervention may offset initially small, but steadily-increasing changes in cardiovascular risk factors for those who eventually make long-term behavioral changes. Future studies should modify the length of the intervention, which might result in larger fitness improvements. While our study has a short length of follow-up, limiting the strength of findings on the mid- to long-term effects of the ZumBeat dance intervention, prospective research should examine participants’ appraisal of the program and physical activity levels in the follow-up period after the intervention.

## 5. Conclusions

A 12-week ZumBeat dance intervention may not suffice to improve cardiorespiratory fitness or substantially improve body composition in sedentary postmenopausal overweight women. However, it shows good feasibility in terms of adherence and safety, helps to improve weight-related quality of life and to reduce sports-related barriers. Future studies are needed to evaluate whether the psychosocial improvements are persistent after the intervention and whether these are transformable into additional health gains.

## Supplementary Materials

The following are available online at www.mdpi.com/2075-4663/4/1/5/s1.
