# Supplementary material for: ZumBeat: Evaluation of a Zumba Dance Intervention in Postmenopausal Overweight Women
Source: Sports (Basel). 2016 Jan 25;4(1):5. doi: 10.3390/sports4010005 (PMC5968936; doi:10.3390/sports4010005)
Supplement: Supplementary file 1 [file sports-04-00005-s001.pdf]

Supplementary Materials: ZumBeat: Evaluation of a Zumba Dance Intervention in Postmenopausal Overweight Women

Anja Rossmeissl, Soraya Lenk, Henner Hanssen, Lars Donath, Arno Schmidt-Trucksäss and Juliane Schäfer

Table S1. Brief description of the neuropsychological test battery.

Acronym	Full Test Name	Brief Description of the Test
IWQOL [1]	Impact of Weight on Quality of Life	Assesses quality of life in overweight populations; 31 items in the categories bodily function, self-esteem, sexual life, public stress and work; scoring on a 5-point scale ranging from "never" to "always true".
MRS [2]	Menopausal Rating Scale	Examines the severity of menopausal symptoms and their influence on quality of life, sensitive to changes over time.
SBB [3]	Sports-Related Situational Barriers	Assesses external and internal situations that dissuade people from the intention to exercise; 13 situations on two subscales (psychological and physical barriers); score ranges between 1 and 4 per item.
BDI-II [4]	Beck Depression Inventory	Assesses severity of perceived symptoms in relation to possible underlying depression with 21 items on a 4-point scale.
ISR [5]	ICD-10-Symptom Rating	Assesses self-rated prevalence and severity of a large spectrum of psychological symptoms with 29 items on a 5-point Likert scale; 5 categories: depression, anxiety, obsessive/compulsive, somatoform, eating disorders, supplementary scale.
I-8 [6]	Impulsivity-8	Assesses the trait "impulsive behavior" on four subscales: urgency, intention, endurance and risk; with 2 items per subscale on a 5-point scale.
FEV [7]	German version of the Dutch Eating Behavior Questionnaire	Fifty-one items assess eating behavior in the subscales dietary restraint (cognitive control), disinhibition and hunger; sum scores are calculated per subscale and compared to norms.

Table S2. Effect of the 12-week ZumBeat dance intervention on cardiorespiratory fitness, anthropometry and psychometry after excluding outliers.

Parameter	Baseline (Mean (SD))	Follow-up (Mean (SD))	Change from Baseline (95% CI)	*p*-Value
VO_{2peak} [1], mL/kg/min	24.7 (3.0)	24.5 (3.0)	−0.2 (−0.8, 0.3)	0.382
HR_{max} [2], bpm	163.6 (16.5)	163.1 (18.4)	−0.5 (−3.7, 2.7)	0.735
Anthropometry				
Percent body fat [3], %	38.6 (8.2)	38.5 (7.3)	−0.1 (−1.3, 1.2)	0.922
Muscle mass [4], kg	30.7 (5.3)	31.0 (5.2)	0.3 (−0.4, 1.1)	0.326
Psychometry				
IWQOL (0 to 100)	–	–	–	–
Total score [4]	82.7 (12.7)	88.9 (9.1)	6.2 (1.8, 10.5)	0.009
Physical function [4]	78.7 (14.0)	83.4 (13.8)	4.7 (−1.5, 11.0)	0.126
Self-esteem [4]	72.8 (24.0)	83.2 (20.2)	10.4 (3.5, 17.4)	0.007
Sexual life [4]	89.4 (23.2)	92.3 (18.8)	2.9 (−0.1, 5.8)	0.054
Public distress [5]	98.2 (4.6)	97.3 (4.7)	−0.9 (−2.9, 1.1)	0.341
Work [6]	93.8 (9.2)	97.4 (5.6)	3.6 (0.1, 7.2)	0.046
MRS [3] (0 to 44)	10.2 (6.4)	9.2 (7.2)	−1.1 (−2.4, 0.2)	0.097
BDI [7] (0 to 63)	5.9 (6.4)	4.5 (5.4)	−1.5 (−4.0, 1.1)	0.235
ISR [7] (0 to 4)	0.5 (0.5)	0.5 (0.4)	0.0 (−0.1, 0.1)	0.453
I-8 (0 to 4)	–	–	–	–
Urgency [4]	2.3 (0.6)	2.1 (0.6)	−0.2 (−0.4, 0.0)	0.035
Endurance [7]	4.2 (0.6)	4.1 (0.5)	−0.1 (−0.3, 0.2)	0.549

Notes: Abbreviations: VO_{2peak}, peak oxygen consumption; HR_{max}, maximum heart rate; IWQOL, Impact of Weight on Quality of Life; MRS, Menopause Rating Scale; BDI, Beck Depression Inventory; I-8, German Version of the UPPS Impulsive Behavior Scale; ISR, ICD-10-Symptom-Rating; SD, standard deviation; CI, confidence interval. [1] After excluding two participants due to failure to reach exertion, available at baseline and follow-up in 11/15 participants; [2] after excluding two participants due to failure to reach exertion, available at baseline and follow-up in 10/15 participants; [3] after excluding two outliers (with an unusually strong decrease in percent body fat and MRS, respectively), available at baseline and follow-up in 12/15 participants; [4] after excluding one outlier (with an unusually strong increase in muscle mass, IWQOL total score, IWQOL physical function, IWQOL self-esteem, IWQOL sexual life and I-8 urgency, respectively), available at baseline and follow-up in 13/16 participants; [5] after excluding three outliers (with an unusually strong increase in IWQOL public distress), available at baseline and follow-up in 11/14 participants; [6] after excluding two outliers (with an unusually strong increase in IWQOL work), available at baseline and follow-up in 12/15 participants; [7] after excluding one outlier (with an unusually strong decrease in BDI, I-8 endurance and ISR, respectively), available at baseline and follow-up in 13/16 participants.

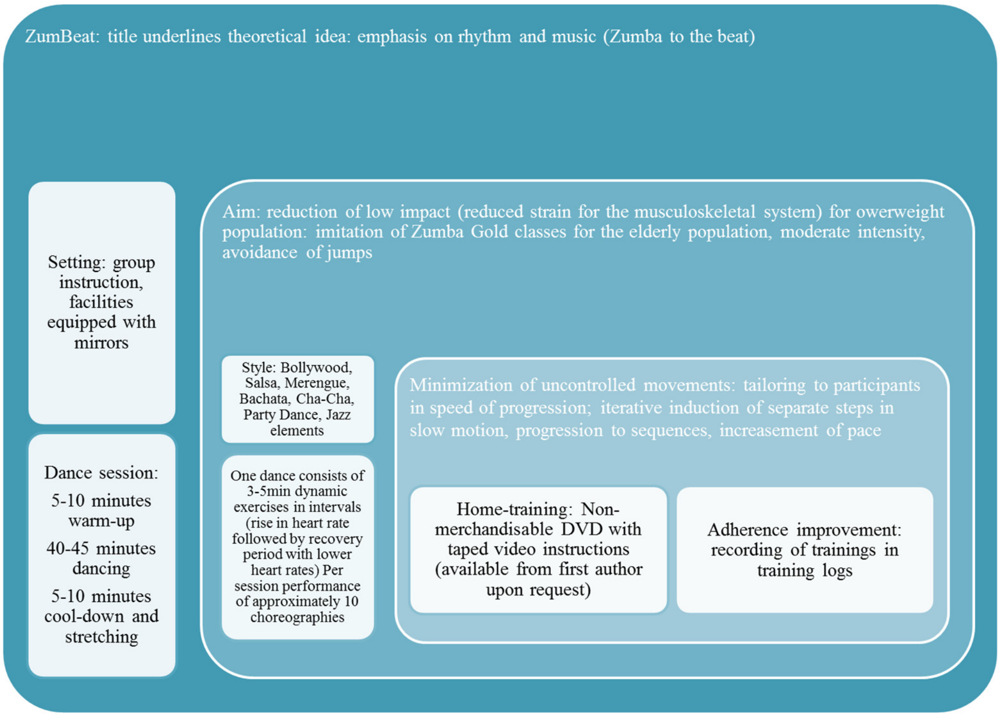

Figure S1. Detailed description of the ZumBeat dance intervention according to the TIDieR checklist [8].

(A)

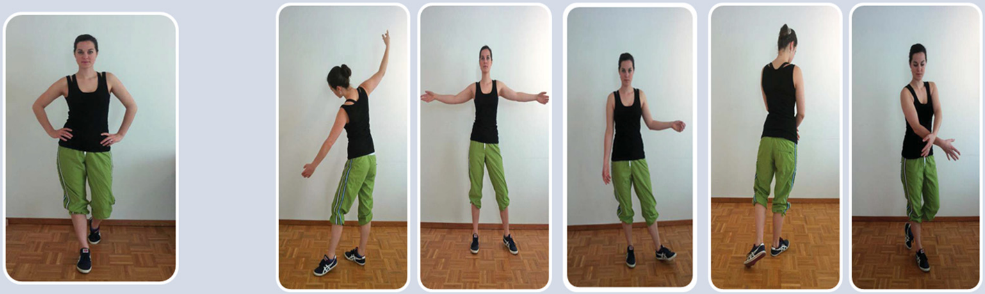

1 2a 2b 3 4 5

Time		Steps
0:00-3:25	Slow motion demonstration of steps	1st step: left forwards - right backwards – cha-cha-cha (small marches to the back) right forwards - left backwards – cha-cha-cha (to the front) (Picture 1)

2nd step: same as 1st step with a 360° turn

3rd step: right side crosses left side with a quick

weight bearing tab, push weight back

to other foot, cha-cha-cha to the left

side, left foot crosses right, push

weight back to right foot, cha-cha-cha

to the left

4th step:

add arms by swinging parallel arm to the front of the body (Picture 2a) and open arms to the side during the cha-cha-cha (Picture 2b)

5th step: turn 360°: start rotation by stepping diagonal with left foot initiating the turn (Picture 3), continue to turn by stepping into the direction of turn with a right foot front and back tab and continue the turn (Picture 4)

6th step: diagonal steps with crossed arms and reach to the side with wide arms during the back tap, turing 360° like that (picture 5)

(B)

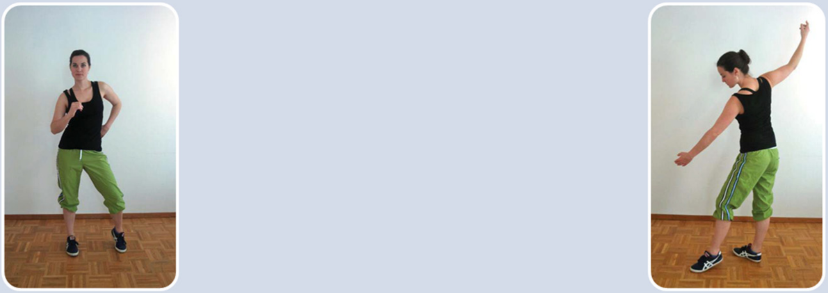

6 7

Time		Steps
3:26-7:03	Combination to the whole dance piece	Right side: step right bearing weight shortly and pushing weight immediately back to the left foot (tab) (short salsa side steps) 4 Left side does the same to the left afterwards (4 repetitions) (Picture 6)

Left side: front tab cha-cha-cha (to the back), right foot tab back cha-cha-cha to the front (3 repetitions with alternating legs)

Initiate a turn step (step #2) with left side, continue with 3 front/back steps/cha-cha-cha (step #1)

Diagonal steps with swinging arms (4 repetitions) begin towards side (step # 3) (Picture 7)

Turn 360° with crossed arms in 4 counts (step # 6), continue with 4 diagonal steps per side, then start from the top and repeat a few more times

Figure S2. Exemplary description of dance Number 4 (cha-cha-cha). (**A**) Steps 1 to 5; (**B**) Steps 6 to 7.

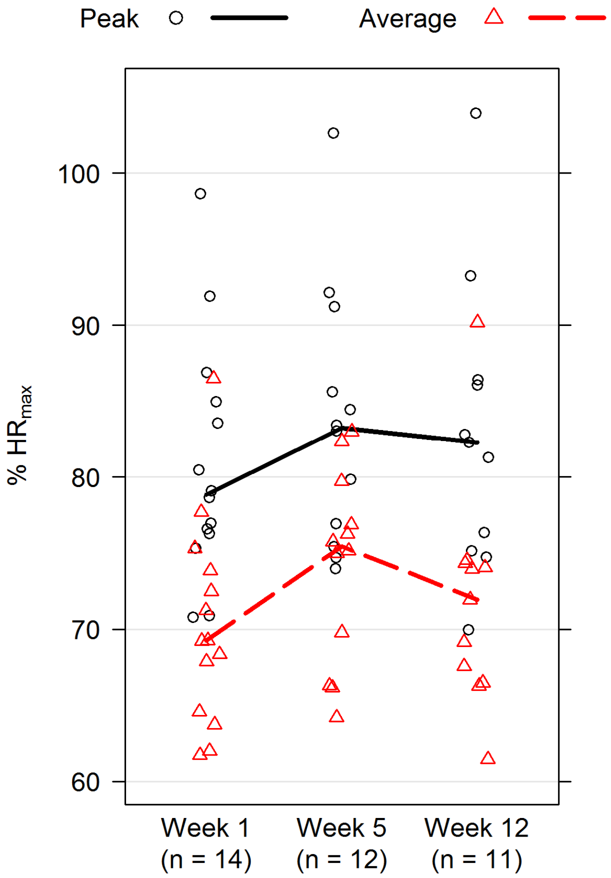

Figure S3. Peak and average heart rate in % of the maximum heart rate (HR_{max}) during three (instructed) training sessions in Weeks 1, 5 and 12. The points denote individual measurements, jittered horizontally to avoid over-plotting. The lines join the medians of the points for the peak and average heart rate in % of the HR_{max}.

References

1. Kolotkin, R.L.; Crosby, R.D.; Kosloski, K.D.; Williams, G.R. Development of a brief measure to assess quality of life in obesity. *Obes. Res.* **2001**, *9*, 102–111.
2. Potthoff, P.; Heinemann, L.A.; Schneider, H.P.; Rosemeier, H.P.; Hauser, G.A. The Menopause Rating Scale (MRS II): Methodological standardization in the German population. *Zentralbl. Gynakol.* **2000**, *122*, 280–286.
3. Krämer, L.; Fuchs, R. Barrieren und Barrierenmanagement im Prozess der Sportteilnahme. Zwei neue Messinstrumente. *Z. Gesundheitspsychol.* **2010**, *18*, 170–182.
4. Beck, A.T.; Steer, R.A.; Ball, R.; Ranieri, W. Comparison of beck depression inventories I and II in psychiatric outpatients. *J. Pers. Assess.* **1996**, *67*, 588–597.
5. Tritt, K.; von Heymann, F.; Zaudig, M.; Zacharias, I.; Sollner, W.; Loew, T. Development of the "ICD-10-Symptom-Rating"(ISR) questionnaire. *Z. Psychosom. Med. Psychother.* **2008**, *54*, 409–418.
6. Kovaleva, A.; Beierlein, C.; Schmitt, M.; Kemper, C.J. Eine Kurzskala zur Messung von Impulsivität nach dem UPPS-Ansatz: Die Skala impulsives Verhalten (I-8). In *GESIS Working Papers*; GESIS: Köln, Germany, 2012; Volume 20.
7. Pudel, D.; Westenhöfer, J. *Fragebogen zum Essverhalten (FEV), Handanweisung*; Hogrefe: Göttingen, Germany, 1989.
8. Hoffmann, T.C.; Glasziou, P.P.; Boutron, I.; Milne, R.; Perera, R.; Moher, D.; Altman, D.G.; Barbour, V.; Macdonald, H.; Johnston, M.; *et al.* Better reporting of interventions: Template for intervention description and replication (TIDieR) checklist and guide. *BMJ* **2014**, *348*, g1687.
